# An Artificial Neural Network Model for Predicting Successful Extubation in Intensive Care Units

**DOI:** 10.3390/jcm7090240

**Published:** 2018-08-25

**Authors:** Meng-Hsuen Hsieh, Meng-Ju Hsieh, Chin-Ming Chen, Chia-Chang Hsieh, Chien-Ming Chao, Chih-Cheng Lai

**Affiliations:** 1 Department of Electrical Engineering and Computer Science, University of California, Berkeley, CA 94720, USA; emersonhsieh@berkeley.edu; 2 Department of Medicine, Poznan University of Medical Science, 61-701 Poznan, Poland; 76519@student.ump.edu.pl; 3 Department of Recreation and Health Care Management, Chia Nan University of Pharmacy and Science, Tainan 71710, Taiwan; 4 Department of Intensive Care Medicine, Chi Mei Medical Center, 901 Chung Hwa Road, Yang Kang City 71044, Taiwan; 5 Department of Pediatrics, China Medical University Children’s Hospital, China Medical University, Taichung 40402, Taiwan; d8336@mail.cmuh.org.tw; 6 Department of Intensive Care Medicine, Chi Mei Medical Center, Liouying District, Tainan 73657, Taiwan; ccm870958@yahoo.com.tw

**Keywords:** predictor, successful extubation, artificial neural network

## Abstract

Background: Successful weaning from mechanical ventilation is important for patients in intensive care units (ICUs). The aim was to construct neural networks to predict successful extubation in ventilated patients in ICUs. Methods: Data from 1 December 2009 through 31 December 2011 of 3602 patients with planned extubation in Chi-Mei Medical Center’s ICUs was used to train and test an artificial neural network (ANN). The input was 37 clinical risk factors, and the output was a failed extubation prediction. Results: One hundred eighty-five patients (5.1%) had a failed extubation. Multivariate analyses revealed that failure was positively associated with therapeutic intervention scoring system (TISS) scores (odds ratio [OR]: 1.814; 95% Confidence Interval [CI]: 1.283–2.563), chronic hemodialysis (OR: 12.264; 95% CI: 8.556–17.580), rapid shallow breathing (RSI) (OR: 2.003; 95% CI: 1.378–2.910), and pre-extubation heart rate (OR: 1.705; 95% CI: 1.173–2.480), but negatively associated with pre-extubation PaO_2_/FiO_2_ (OR: 0.529; 95%: 0.370–0.750) and maximum expiratory pressure (MEP) (OR: 0.610; 95% CI: 0.413–0.899). A multilayer perceptron ANN model with 19 neurons in a hidden layer was developed. The overall performance of this model was F_1_: 0.867, precision: 0.939, and recall: 0.822. The area under the receiver operating characteristic curve (AUC) was 0.85, which is better than any one of the following predictors: TISS: 0.58 (95% CI: 0.54–0.62; *p* < 0.001); 0.58 (95% CI: 0.53–0.62; *p* < 0.001); and RSI: 0.54 (95% CI: 0.49–0.58; *p* = 0.097). Conclusions: The ANN performed well when predicting failed extubation, and it will help predict successful planned extubation.

## 1. Introduction

A significant percentage of intensive care unit (ICU) patients require endotracheal intubation [1]. Prolonged ventilatory support increases the risk of complications, such as ventilation-associated pneumonia, and could be associated with higher in-hospital mortality and greater post-discharge mortality, healthcare utilization, and healthcare costs [2]. Thus, extubation of ventilated patients as early as possible after respiratory stabilization is desirable [3]. To reduce the risk of prolonged ventilatory support, it is crucial to determine the appropriate time for weaning a patient from mechanical ventilation [4] because extubation failure might occur in premature extubation. Standard clinical practice is to extubate based on a comprehensive assessment that considers a patient’s clinical condition, arterial blood gas results, ventilator settings, and weaning profiles [5]. However, extubation failure often occurs (~19% reintubation required) even after the comprehensive assessment [6]. This suggests that the ability of clinicians to predict successful extubation is limited; a more powerful tool is required to help determine the optimal time to extubate [7].

Outcome prediction models using artificial neural networks (ANNs) and multivariable logistic regression analyses have recently been developed in many areas of healthcare research [8,9]. Artificial neural networks are computer-based algorithms that mimic the habits and structures of neurons. They have also been successfully used to predict mortality in trauma patients [10]. Recently, ANNs have been introduced to predict extubation outcomes, but findings vary by study [11,12]. The main reasons for poor outcome predictions might be because of differences in clinical input data. It was aimed to construct an ANN model for clinicians making extubation decisions.

## 2. Materials and Methods

### 2.1. Patients and Setting

This study retrospectively analyzed 3602 adult patients with planned extubation in eight ICUs of Chi-Mei Medical Center from December 2009 through December 2011. All of them were enrolled in a prospective observational study [13]. All patients were separated into two groups: a successful extubation group and a failed extubation group. Patients who remained extubated after 72 h were classified as having a successful extubation, even if they required reintubation later during the same hospitalization [14,15]. In contrast, patients who needed reintubation within 72 h after a planned extubation were classified as having a failed extubation. Patients who died within 72 h of extubation are also considered as going through an extubation failure. Noninvasive ventilation (NIV) may be considered to rescue extubation failure [16,17]. Patients who withstood NIV without reintubation for more than 72 h after extubation were classified as having a successful extubation. There were 161 patients treated with NIV, and 29 patients needed reintubation within 72 h. Demographic and clinical information, laboratory results, comorbidities, and the severity scores of all patients were collected. Chi-Mei Medical Center’s Institutional Review Board approved the study protocol (IRB no. 10706-009).

### 2.2. Constructing Training Data Set

All features were extracted from the original dataset. The data of all patients was normalized to have an overall mean of 0 and a standard deviation of 1. After data processing, there were 37 input features, each of which were chosen for their wide availability in ICUs, and two outputs, each of which represented a prediction of successful or failed extubation.

### 2.3. Data Description

The entire data set was comprised of 3602 data points. In both the training and test data sets, the positive class was dominant: 3416 of 3602 (94.8%) patients had a successful extubation. The ratio between successfully and unsuccessfully extubated patients was 1:18.47. The data were split into training and test sets at approximately a 9:1 ratio, which was chosen in accordance with other ANN research [18]. The 3242 data points were randomly allocated to the train set and 360 data points were randomly allocated to the test set.

### 2.4. Algorithm and Training

A multilayer perceptron (MLP) neural network was used to train the data. *K*-fold cross-validation with a *k* value of 10 was used over 10 epochs to select the best-performing hyperparameters, optimizers, and loss function. The three-layered model consists of one input layer with 37 dimensions, a hidden layer of 19 dimensions, and an output layer of 2 dimensions. The network was trained using stochastic gradient descent with a mini-batch size of 1. The network was optimized using Adam with default parameters as described by Kingma et al. [9]. The neural network was trained for 60 epochs. The Scaled Exponential Linear Unit (SeLU) activation function was used at each layer, and Softmax was used at the output layer [15]. A 20% dropout rate (a simple way to prevent neural networks from overfitting) was applied to the input layer and a 50% dropout rate was applied to output layer [19]. The categorical cross-entropy error function for binary classifiers was used as the loss function. Each data point was weighted based on its outcome ratio; this was done to ensure that the output of the neural network was not heavily skewed toward the dominant class.

The software was implemented using Python 3.6.5 [20] with the scikit-learn library (version 0.19.1) [21] and the Tensorflow framework (version 1.8.0) [22].

### 2.5. Statistical Analyses

Mean values, standard deviations, and group sizes were used to summarize the results for continuous variables. The differences between the successful and failed extubation groups at hospital discharge were examined using univariate analysis with a Student’s *t* test and a *χ^2^* test. Significance was set at *p* < 0.05. Predetermined variables, or those significantly associated with successful extubation in univariate analysis (*p* < 0.05), were tested for interaction using multivariate logistic regression analysis. Odds ratios (ORs) and 95% confidence intervals (CIs) were calculated. SPSS 24.0 for Windows (SPSS, Inc., Chicago, IL, USA) was used for all statistical analyses.

Because the data distribution was unbalanced, accuracy was not a reliable measurement of predictor performance [23]. Instead, the weighted averaged recall (sensitivity), precision (positive predictive value [PPV]), and F_1_ scores (harmonic mean of sensitivity and precision) were used to measure ANN performance. The value of ideal recall, precision, and F_1_ scores = 1 [24]. All three scores were calculated for the test set and for all data.

The ANN performance was also measured using the area under the receiving operating characteristic (ROC) curve. The area under the ROC curve (AUC) of the neural network was compared against the AUC of variables that had significantly different outcomes. The AUC was also compared against the ideal value of 1 [25].

To ensure that it is the ANN and not the individual variables that improve the prediction, the ROC of the ANN was compared with that of a composite score created from relevant variables. To create a composite score that was representative of individual variables, principal component analysis (PCA) was first performed on the significant variables. A composite score was then created by the results of the multivariate analysis. The variable weightings in the composite score were based on its correlation with the first principal component.

## 3. Results

### 3.1. Demographic Features of Patients

Table 1 shows the demographic and clinical characteristics of the sample of ICU patients with planned extubation. Of the 3602 patients included in the study, 50.9% were male and 49.1% were female. Patients with extubation failure were older than the successful extubation group (*p* < 0.001). In addition, patients with extubation failure had higher Acute Physiology and Chronic Health Evaluation (APACHE) II scores (18.9 ± 7.0 vs. 16.2 ± 7.4) and therapeutic intervention scoring system (TISS) scores (29.3 ± 7.5 vs. 27.1 ± 7.8) than the successful extubation group (both *p* < 0.001). Regarding weaning parameters, there is a significant difference in the TISS score, maximum expiratory pressure (MEP), and rapid shallow-breathing index (RSI) between the patients in the failed and successful extubation groups (all *p* < 0.05). Overall, failed extubation patients had longer duration of mechanical ventilation (MV) uses (140.8 ± 145.8 h vs 106 ± 126.9 h) than patients with successful extubation (*p* = 0.002).

Multivariate analyses showed that failed extubations were positively associated with TISS scores, chronic hemodialysis, RSI, and pre-extubation heart rate, but negatively associated with pre-extubation PaO_2_/FiO_2_ and MEP (Table 2).

### 3.2. Results of Artificial Neural Networks (ANN)

The overall performance of the ANN model was shown in Table 3. The weighted k-fold accuracy of the ANN (*k* = 10) was 0.94.

**Figure 1:**
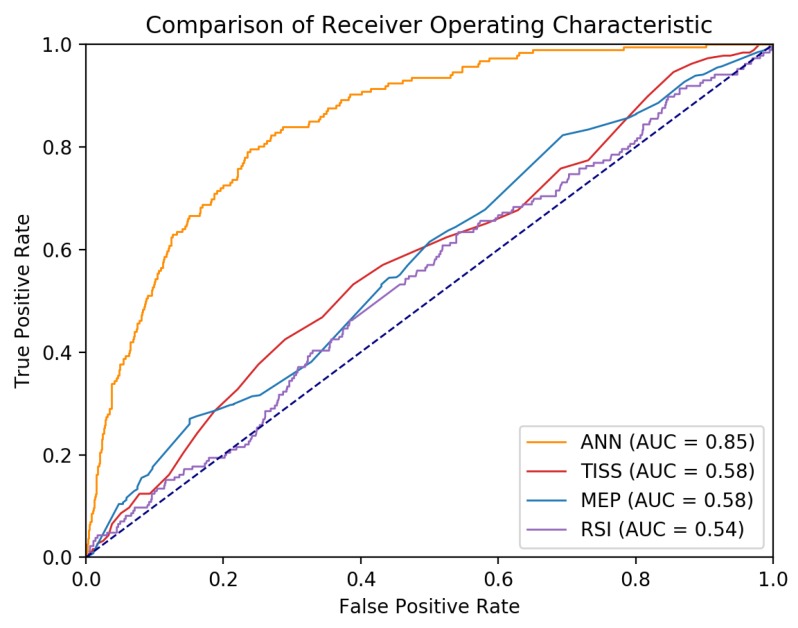
Receiver operating characteristic curve of artificial neural network (ANN), Therapeutic Intervention Scoring System (TISS), maximum expiratory pressure (MEP), and rapid shallow breathing index (RSI) for predicting successful weaning in ICU patients.

Figure 1 shows the ROC curve of the ANN, TISS, MEP, and RSI on all patient data. The AUC in the test set of the ANN model was 0.85 (95% CI: 0.82–0.87, *p* < 0.001), which was better than any one of the following predictors: 0.58 (95% CI: 0.54–0.62, *p* < 0.001) for TISS, 0.58 (95% CI: 0.53–0.62, *p* < 0.001) for MEP, and 0.54 (95% CI: 0.49–0.58, *p* = 0.097) for RSI. Whether there was a significant difference between the ANN and other variables was determined using the DeLong test on the AUC [26]. There is a significant difference between the AUC for the ANN and the AUC for TISS (*z* = 10.71, *p* < 0.0001), MEP (*z* = 10.95, *p* < 0.0001), and RSI (*z* = 12.52, *p* < 0.0001).

The weight of each variable in the composite score used as a point-of-comparison are summarized in Table 4.

**Figure 2:**
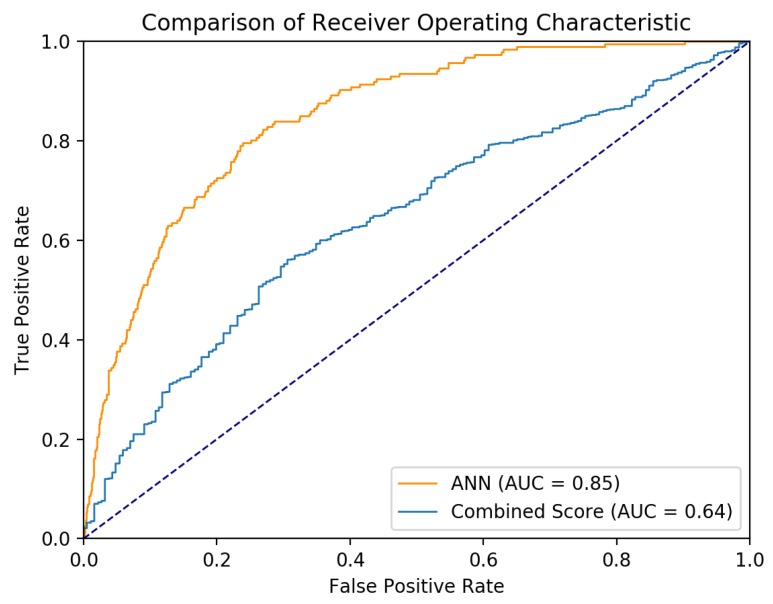
Receiver operating characteristic curve of artificial neural network (ANN) and the composite score created from significant variables.

Figure 2 shows the ROC curve of the ANN and the composite score. The AUC of the combined score was 0.64 (95% CI 0.60–0.68, *p* < 0.001). The AUC of the ANN was significantly better than the AUC of the combined score (*z* = 8.79, *p* < 0.0001).

## 4. Discussion

It was found that a neural network model is a good predictor for successful extubation. While other weaning parameters, such as tidal volume, frequency, minute ventilation, MEP, and RSI, are used to help assess the weaning process, they did not yield a high degree of accuracy in predicting extubation outcomes [27,28,29,30]. It was also found that the predictive performance of ANN was better than those of RSI and MEP. This is consistent with a previous study [11] which reported that the proposed ANN yielded better discrimination for predicting successful extubation than did the RSI and PI max. Also, it was found that the ANN yielded a better performance than a composite score based on significant variables created using PCA. Moreover, the ANN in this study was created and trained based on the data of 3602 ready-to-wean patients, far more than in Kuo et al. [11]. Finally, the ANN algorithm provided useful information about the optimal time to extubate.

Several other studies have tried to find appropriate predictors of successful weaning and presented different findings. In other studies, the factors that predicted failed weaning were older age, pulmonary cause of intubation, and lower mean arterial pressure [13,31]. The prediction of successful extubation have also been reported: being female and low blood urea creatinine [32]; MIP and arterial carbon dioxide tension (PaCO_2_) [33]; and respiratory rate, RSI, MIP, and APACHE II scores [27]. All these factors were included in the current ANN algorithm; thus, it should provide an accurate prediction based on comprehensive information.

Previous ANNs were usually developed using proprietary software such as Statistica (TIBCO Software Inc., San Francisco, CA, USA) and SPSS. The free and open source Tensorflow framework was used to create our ANN. Tensorflow has a fast update cycle and frequently incorporates newer neural network configurations. In contrast, SPSS has a slow update cycle and fewer configurations. For instance, SPSS 25 does not have the Adam optimizer, which was used in the present ANN.

This study has some limitations. First, the rate of extubation failure was particularly low in this study (5%) compared with rates in the literature (about 15%). In the present study, the final decision to extubate was made by the intensivists treating the intubated patients. Thus, it was possible that they did not follow the weaning and extubation protocol. Second, delayed extubation might have occurred in this study. Third, the dataset used for this project was from December 2009 through December 2011; thus, the rapid advancements in sedation practices, delirium awareness, early mobility, anesthesia and pain management, and ventilator capacity over the last decade might be a significant confounder to the utility of this work. Fourth, the selection of variables for the model was based only on the widespread availability of these data. This “availability” may depend from the type and habits of each ICU. The findings may not be generalized to other ICUs. Finally, patients who needed reintubation or died within 72 h after a planned extubation were classified as having a failed extubation. As NIV can postpone the need for reintubation, a period of 7 days after extubation is required for a more accurate definition of extubation failure when NIV is of broad use [34].

## 5. Conclusions

An extubation strategy for all ventilated ICU patients should be thoroughly planned. The present study shows the parameters used to predict a successful planned extubation using an ANN. Failed extubations were positively associated with TISS, RSI, pre-extubation heart rate, and chronic hemodialysis, but negatively associated with MEP and pre-extubation PaO_2_/FiO_2_. Furthermore, this present ANN model efficaciously predicted successful planned extubations in ICU patients.

## Figures and Tables

**Table 1:** Demographic and clinical characteristics of 3602 intensive care unit (ICU) patients with planned extubation.

Variable	Successful Extubation	Failed Extubation	*p*
	*n* = 3417 (94.9%)	*n* = 185 (5.1%)	
Age (years)	63.9 ± 16.5	68.1 ± 14.6	<0.001
Male	1729 (50.6%)	106 (57.3%)	0.426
BMI (kg/m^2^)	23.7 ± 4.5	23.2 ± 4.5	0.144
APACHE II	16.2 ± 7.4	18.9 ± 7.0	<0.001
TISS Scale	27.1 ± 7.8	29.3 ± 7.5	<0.001
Glasgow coma scale	12.1 ± 3.5	11.1 ± 3.8	0.001
Comorbidities			
Cardiovascular accident	635 (18.6%)	45 (24.3%)	0.052
Chronic lung disease	264 (7.7%)	21 (11.4%)	0.075
Chronic hemodialysis	314 (9.2%)	105 (56.8%)	<0.001
Chronic liver disease	76 (2.2%)	3 (1.6%)	0.586
Diabetes	999 (29.2%)	71 (38.4%)	0.008
Old stroke	866 (25.3%)	56 (30.3%)	0.135
Active cancer	755 (22.1%)	28 (15.1%)	0.025
Pre-extubation data			
FiO_2_	27.4 ± 3.5	28.0 ± 3.7	0.029
Pressure support level	9.2 ± 1.5	9.2 ± 1.6	0.840
PEEP	5.1 ± 0.5	5.2 ± 0.6	0.028
Minute ventilation	7.8 ± 2.6	7.5 ± 2.4	0.075
Pulse rate	86.6 ± 16.2	87.9 ± 17.3	0.236
Mean arterial pressure	96.5 ± 16.2	94.7 ± 18.1	0.184
Respiratory rate	16.7 ± 5.1	18.0 ± 5.3	0.001
pH	7.441 ± 0.054	7.446 ± 0.051	0.279
PaCO_2_	37.6 ± 6.2	38.5 ± 6.0	0.057
PaO_2_	105.9 ± 41.6	94.9 ± 27.4	<0.001
PaO_2_/FiO_2_	361.0 ± 101.0	329.3 ± 94.1	<0.001
Hemoglobin	11.3 ± 1.9	10.7 ± 1.8	<0.001
Hematocrit (%)	34.2 ± 6.7	32.4 ± 6.7	0.001
Blood urea nitrogen	25.1 ± 21.3	32.9 ± 31.8	0.002
Creatinine	1.7 ± 2.1	1.9 ± 2.1	0.200
Sodium	139.1 ± 4.6	138.8 ± 5.1	0.370
Potassium	3.8 ± 0.5	3.9 ± 0.5	0.398
Calcium	7.9 ± 0.9	8.0 ± 0.9	0.598
Phosphate	3.4 ± 1.5	3.3 ± 1.7	0.812
Albumin	2.8 ± 0.6	2.7 ± 0.6	0.074
Weaning parameters			
RSI	52.8 ± 29.9	62.8 ± 33.2	<0.001
MIP	37.9 ± 14.1	34.9 ± 13.0	0.008
MEP	61.0 ± 29.4	52.6 ± 26.7	<0.001
Ventilator use duration (*h*)	106.0 ± 126.9	140.8 ± 145.8	0.002

*χ*^2^ test, and *t*-test comparing survivors and non-survivors overall; Data are presented as mean ± standard deviation or *n* (%); APACHE = Acute Physiology and Chronic Health Evaluation; BMI = body mass index; TISS = Therapeutic Intervention Scoring System; ICU = intensive care unit; RSI = Rapid Sallow-breathing Index; MIP = Maximum negative Inspiratory Pressure; MEP = Maximum Expiratory Pressure.

**Table 2:** Significant predictors of the failed extubation of all planned extubation patients.

Variable	OR	95% CI	*P* *	OR	95% CI	*P* **
Age (years)	1.107	1.007–1.027	0.001			
APACHE II	1.046	1.027–1.066	<0.001			
TISS Scale	1.036	1.017–1.055	<0.001	1.814 ^#^	1.283–2.563	0.001
Glasgow coma scale	0.930	0.894–0.967	<0.001			
**Comorbidities**						
Chronic hemodialysis	12.970	9.483–17.740	<0.001	12.264	8.556–17.580	<0.001
Diabetes	1.507	1.110–2.045	0.008			
Active cancer	0.629	0.417–0.948	0.027			
Ventilator use duration (*h*)	1.002	1.001–1.003	<0.001			
**Weaning parameter**						
RSI	1.008	1.004–1.012	<0.001	2.003 ^%^	1.378–2.910	<0.001
MIP	0.983	0.970–0.995	0.008			
MEP	0.989	0.983–0.995	<0.001	0.610 ^@^	0.413–0.899	0.013
**Pre-extubation data**						
Pulse rate	1.014	1.005–1.023	0.003	1.705 *	1.173–2.480	0.005
PaO_2_/FiO_2_	0.997	0.995–0.998	<0.001	0.529 ^&^	0.373–0.750	<0.001
Hemoglobin	0.832	0.765–0.904	<0.001			
Hematocrit (%)	0.961	0.939–0.984	0.001			
BUN	1.012	1.007–1.017	<0.001			

^#^: TISS ≥ 28.5, AUC ≈ 0.6, *p* < 0.001; ^%^: RSI ≥ 49.95, AUC ≈ 0.6, *p* < 0.001; ^@^: MEP < 39 cm H_2_O, AUC ≈ 0.6, *p* < 0.001; Pulse rate ≥ 102.5 /min, AUC ≈ 0.6, *p* = 0.026; ^&^: PaO_2_/FiO_2_ ≤ 323.25, AUC ≈ 0.6, *p* < 0.001; * Value for univariate analysis; ** Value for multivariate analysis;

**Table 3:** Weighted average F_1_, precision, and recall values for all data sets.

	Test Set (*n* = 37)	All Patients (*n* = 307)
F_1_	0.871	0.867
Precision	0.957	0.939
Recall	0.808	0.822

**Table 4:** Weighing of each variable in the composite score.

Variable	Weighting
Age (years)	−0.474
APACHE II	−0.75
TISS Scale	−0.286
Glasgow coma scale	0.566
Comorbidities	
Chronic hemodialysis	−0.289
Diabetes	−0.022
Active cancer	0.027
Ventilator use duration (*h*)	−0.611
Weaning parameter	
RSI	−0.005
MIP	0.238
MEP	0.353
Pre-extubation data	
Pulse rate	0.066
PaO_2_/FiO_2_	0.097
Hemoglobin	0.692
Hematocrit (%)	0.643
BUN	−0.033

## Supplementary Materials

The following are available online at http://www.mdpi.com/2077-0383/7/9/240/s1, Supplementary 1: The artificial neural network model used in this study and a spreadsheet document that describes the variables for each input feature are attached. Supplementary 2: The neuron weights of the neural network are provided in the HDF5 format and can be imported into a Keras/Tensorflow model with the configuration described in the method section of this study. The neural network is trained on standardized and unity-based normalized data.
